# Screening of Endophytic Antagonistic Bacteria in Wheat and Evaluation of Biocontrol Potential against Wheat Stripe Rust

**DOI:** 10.3390/plants13101366

**Published:** 2024-05-14

**Authors:** Ainisai Saimi, Qiqi Zhang, Qi Liu, Guangkuo Li, Haifeng Gao, Jing Chen

**Affiliations:** 1 Key Laboratory of the Pest Monitoring and Safety Control of Crops and Forests of the Xinjiang Uygur Autonomous Region, College of Agronomy, Xinjiang Agricultural University, Urumqi 830052, China; anisa1010@163.com (A.S.); 18999715375@163.com (Q.Z.); chenj@xjau.edu.cn (J.C.); 2 Key Laboratory of Prevention and Control of Invasive Alien Species in Agriculture & Forestry of the North-Western Desert Oasis (Co-Construction by Ministry and Province), Ministry of Agriculture and Rural Affairs, Urumqi 830052, China; 3 Institute of Plant Protection, Xinjiang Academy of Agricultural Science/Key Laboratory of Integrated Pest Management on Crop in Northwestern Oasis, Ministry of Agriculture and Rural Affairs, Urumqi 830091, China; lgk0808@163.com (G.L.); xghf20044666@163.com (H.G.)

**Keywords:** wheat stripe rust, endophytic bacteria, antagonistic effect, biological control

## Abstract

Wheat stripe rust is globally one of the most important diseases affecting wheat. There is an urgent need to develop environmentally safe and durable biological control options to supplement the control that is achieved with breeding and fungicides. In this study, endophytic bacteria were isolated from healthy wheat through the tissue separation method. Antagonistic endophytic bacteria were screened based on the control effect of urediniospore germination and wheat stripe rust (WSR). The taxonomic status of antagonistic strains was determined based on morphological, physiological, and biochemical characteristics and molecular biological identification (16S rDNA and *gyrB* gene sequence analysis). Finally, the potential growth-promoting effect of different concentrations of antagonists on wheat seedlings and the biological control effect of WSR were studied. A total of 136 strains of endophytic bacteria belonging to 38 genera were isolated. *Pseudomonas* was the most common bacterial genus, with 29 isolates (21%). The biological control effect of different isolates was assessed using an urediniospore germination assay. The isolate XD29-G1 of *Paenibacillus polymyxa* had the best performance, with 85% inhibition of spore germination during primary screening. In the deep screening, the control effect of XD29-G1 on wheat stripe rust was 60%. The antagonist XD29-G1 promoted the germination of wheat seeds and the growth of wheat seedlings at a solution dilution of 10^−7^ cfu/mL. The pot experiment results showed that different dilution concentrations of the strain had different levels of antibacterial activity against WSR, with the concentration of 10^−1^ cfu/mL having the best control effect and a control efficiency of 61.19%. XD29-G1 has better biological control potential against wheat stripe rust.

## 1. Introduction

Wheat stripe rust is a fungal disease caused by *Puccinia striiformis* f. sp. *tritici* (*Pst*). Due to its high frequency, wide region, and serious loss, it has historically caused significant yield losses worldwide. It has become the greatest biotic constraint to wheat production in the twenty-first century, resulting in annual losses of one billion dollars worldwide [1,2]. China is the largest wheat producer, and it is the largest stripe rust endemic region in the world [3,4]. When the disease is serious, the national wheat yield can be reduced by 50~60%, or the harvest is even lost, which seriously endangers China’s food security [5,6].

At present, the main control methods for wheat stripe rust (WSR) domestically and abroad are chemical control, breeding resistant cultivars, timely planting, crop rotation, cultivar mixture utilization, and appropriate fertilization. However, the virulence of WSR varies frequently and produces new virulent species, which leads to the breakdown of resistance genes and the periodical prevalence of WSR [7]. The selection of resistant cultivars is one of the key measures to control wheat stripe rust. However, the high variability of WSR and the large planting of single cultivars lead to the loss of resistance of wheat cultivars, and the resistance of some new cultivars is easily overcome by a new virulent species [8]. Chemical control with fungicides is the second main control option for stripe rust. Dependency on fungicides and prolonging the service time of fungicides have always been the main control measures for various plant diseases. With long-term usage of fungicides, pathogens can easily develop drug resistance [9]. Therefore, how to effectively control the occurrence of *Pst* is of great practical significance to ensure the safe production of wheat.

Beneficial plant endophytes (including endophytic fungi, endophytic bacteria, and actinomycetes), as a kind of microbial resource existing in plants, have been proven to be effective in improving crop yield and resistance to pests and diseases [10,11]. According to the literature, most endophytic bacteria belong to the genera *Bacillus*, *Streptomyces*, and *Pseudomonas* [12,13,14]. Endophytic species’ diversity depends on the diversity of plant and host species, as well as the selectivity of different parts of the host [15]. For example, Chen [16] isolated 267 strains of endophytic bacteria from the root, stem, and leaf tissues of healthy tobacco. The isolates were classified into 21 genera through 16S rRNA sequence homology comparison, where *Bacillus* was the dominant genus of culturable endophytic bacteria. These endophytes of different species have various biological functions, and they can exert multiple influences on plant growth promotion and stress resistance through their own metabolism and growth, which is of great significance for microbial diversity and the development of strains with specific functions.

Endophytic bacteria have beneficial effects on host plants, including nitrogen fixation, plant growth promotion, producing plant hormones, and phosphorus dissolving [17,18]. Nejad [19] found that endophytic bacteria isolated from plant tissues not only promoted seed germination and seedling growth of rape and tomato but also significantly reduced the symptoms of *Verticillium wilt* and *Fusarium wilt*. Wang [20] discovered that the bacterial suspension of three strains of *Paenibacillus polymyxa* had an 83.46%, 49.02%, and 68.31% control effect on root rot of Salvia miltiorrhiza, and that after inoculation with fermentation broth, plant height increased by 29.77%, 32.60%, and 27.64%, respectively. The fresh weight of stems and leaves increased by 18.76%, 30.57%, and 28.42%, respectively. The number of branches increased by 89.12%, 76.53%, and 112.59%, and the fresh weight of roots increased by 88.82%, 122.49%, and 144.78%, respectively.

Studies have shown that plant endophytes have a good control effect on various pathogenic microorganisms and insect pests. Biological control of wheat rust disease has been reported at home and abroad. For example, Kiani [21] found that endophytic bacteria isolated from stripe-rust-resistant varieties had the potential to inhibit urediniospore germination of wheat stripe rust both in vitro and in plant experiments. The endophytic bacteria *Paneibacillus xylanexedens* 7A and *Bacillus megaterium* 6A induced wheat resistance to wheat stripe rust, and the control effects were 61% and 65%, respectively. Pang [22] studied the population diversity of endophytic bacteria in wheat screened out from the isolated endophytic bacteria strains with significant effects on wheat growth promotion, disease prevention, and yield increase, and studied their mechanisms of disease prevention, growth promotion, and yield increase. However, the screening of endophytic bacteria of different winter wheat varieties in Xinjiang and the use of strains with significant biocontrol potential to control wheat stripe rust have not been reported. At present, the main research on wheat stripe rust in Xinjiang focuses on physiological species identification [23,24], over-summering regions [25], resistance gene detection [26], deep learning recognition of disease [27], hyperspectral remote sensing [28,29,30], population genetic structures [31], and the minimum detection limit [32].

*Paenibacillus polymyxa* is an important rhizosphere growth-promoting bacterium. It can be used as a microbial fertilizer to promote crop growth and increase yield, and it can be used as a biological agent to control a variety of plant diseases safely and with no environmental pollution [33,34]. Treating wheat seedlings with *Paenibacillus polymyxa* CCM1465 and 92 fermentation solution and its metabolites, exopolysaccharides, can improve the mitotic index of wheat seedlings’ root cells and effectively promote wheat growth [35]. It has been reported that *Paenibacillus polymyxa* is involved in inducing disease resistance in plant systems. Xu [36] found that Sneb1462 could induce the production of ROS in wheat at the early stage of the infection process of wheat leaf rust, participate in the production process of systemic resistance, and enhance its disease resistance. Mei [37] found that the CFO5 treatment of *Paenibacillus polymyxa* could induce the accumulation of H_2_O_2_ and phenol in the early stage after the inoculation of *Fusarium wilt*, inhibit the invasion of *Fusarium wilt*, and increase the yield of tomato. Kim [38] used *Paenibacillus polymyxa* APEC for biological control of apple anthracnose, and the bacteriostasis rate was up to 83.6%. It can be seen that *Paenibacillus polymyxa* has promising applications in agricultural disease management. However, there are no reports on the use of *Paenibacillus polymyxa* in promoting and controlling WSR.

Therefore, in this study, endophytic bacteria were isolated from different tissues of winter wheat in Xinjiang, and the biocontrol bacteria with the best antibacterial activity were selected. Through physiological, biochemical, and molecular methods, we identified the characteristics and taxonomic statuses of biocontrol strains. We investigated the potential growth-promoting effects of different dilution concentrations of antagonists on wheat seedlings, as well as their biocontrol effectiveness against WSR. This study provides the basis for further development and utilization of endophytic bacteria in wheat and excellent strain resources for the biological control of wheat stripe rust.

## 2. Results

### 2.1. Isolation of Endophytic Bacteria from Wheat

A total of 136 strains of endophytic bacteria were isolated from different tissues (leaves, stems, and roots) of different wheat cultivars. This indicates that there was a large number of endophytic bacteria and abundant species in wheat. We obtained 67 endophytic bacterial isolates from the roots, 54 isolates from the stem, and 15 isolates from the leaves (Table 1). The highest number of endophytic bacteria was isolated from the roots, followed by the stems and the leaves.

### 2.2. Diversity Analysis of Endophytic Bacteria

The 16S rDNA sequence information for all endophytic bacteria was obtained through a 16S rDNA sequence analysis of 136 endophytic bacteria (Appendix A Table A1). According to the obtained 16S rDNA sequence identification information, 136 endophytic bacteria were identified as belonging to 38 genera (Table 2). *Pseudomonas* sp. was the dominant bacterial genus, accounting for 21% of the total strains. *Microbacterium* sp. and *Paenibacillus* sp. followed, accounting for 10% (Appendix A Table A2).

From the diversity of endophytic bacteria in different tissues of wheat, the highest Shannon diversity index of wheat stems was 2.710, followed by the root (2.697) and the leaf (2.079). This indicated that the species diversity was higher in stems than in roots and leaves. The Simpson diversity indexes results showed that the Simpson diversity index of the root and stem of wheat were 0.913 and 0.915, respectively. The Simpson diversity index of leaves was 0.896, which was relatively low. In addition, the Simpson diversity index of overall endophytic bacteria in wheat was 0.921, indicating that, overall, endophytic bacteria in wheat also had high diversity (Table 2).

According to the diversity of endophytic bacteria in different wheat cultivars, Xindong No.41 had the highest Shannon diversity index at 2.754, followed by Xindong No.26 (2.441) and Xindong No.9 (2.168). It could be seen that Xindong No.41 had the highest Shannon diversity. The Simpson diversity index results showed that the Simpson diversity index of Xindong No.26 was the highest, as it reached up to 0.978. It was followed by Xindong No.41, and Simpson’s diversity index was 0.963, which is close to 1, indicating that there was high diversity among different wheat cultivars (Table 3).

### 2.3. Screening of Antagonistic Endophytic Bacteria

#### 2.3.1. Determination of Inhibitory Effect of Endophytic Bacteria on the Germination of Urediniospores

In total, 111 strains of endophytic bacteria were activated from 136 strains to determine the inhibition of urediniospores germination of *Pst* (Appendix A Table A3). Nine endophytic bacteria were screened that had a good inhibitory effect on the germination of urediniospores. These nine strains (XD29-Y1, XD29-Y2, XD5-Y2, XD5-J1, XD14-J3, XD41-J8, XD5-G7, XD29-G1, and XD22-G3) were the most significant endophytic bacteria in different tissues of wheat. The culture solution and the centrifuged supernatant of the culture solution of nine endophytic bacteria had different degrees of inhibition on the germination of urediniospores. The nine strains showed inhibition efficacy greater than 70% in both types of bacterial formulations. However, three strains (XD5-J1, XD5-G7, and XD29-G1) had a strong inhibitory effect on the germination of urediniospores, with an inhibitory rate greater than 85%, with the endophytic bacteria XD5-J1 showing a higher inhibition rate. The inhibition rates of the culture solution and the centrifuged supernatant of the culture solution were 92.71% and 87.85%, respectively.

**Figure 1:**
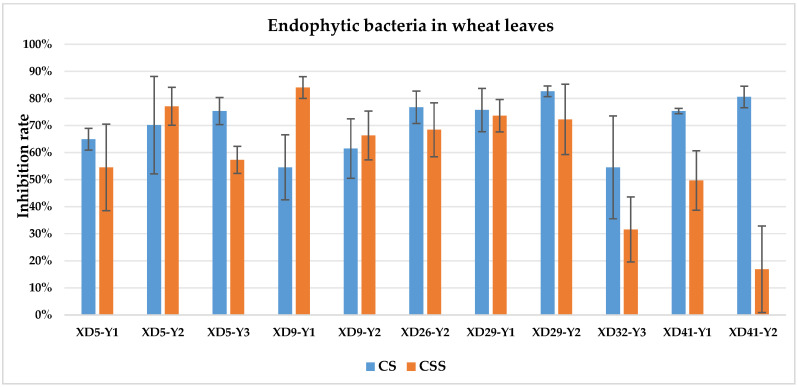
Inhibition of germination of urediniospores by endophytic bacterial strains isolated from wheat leaf. Note: CS: culture solution; CSS: centrifuged supernatant of the culture solution.

**Figure 2:**
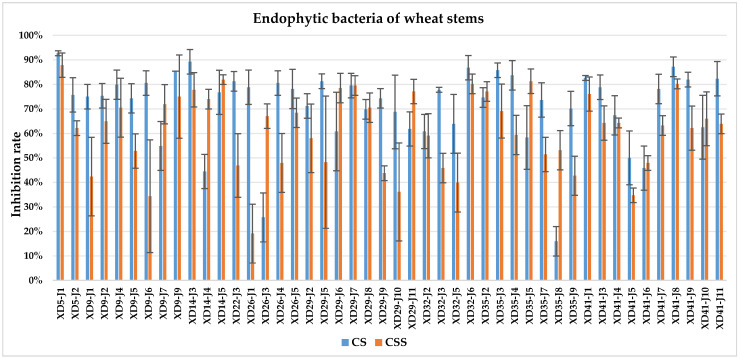
Inhibition of germination of urediniospores by endophytic bacterial strains isolated from wheat stem.

**Figure 3:**
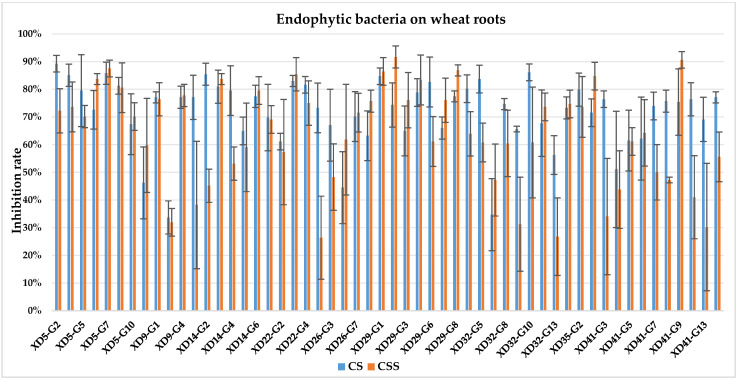
Inhibition of germination of urediniospores by endophytic bacterial strains isolated from wheat root.

**Figure 4:**
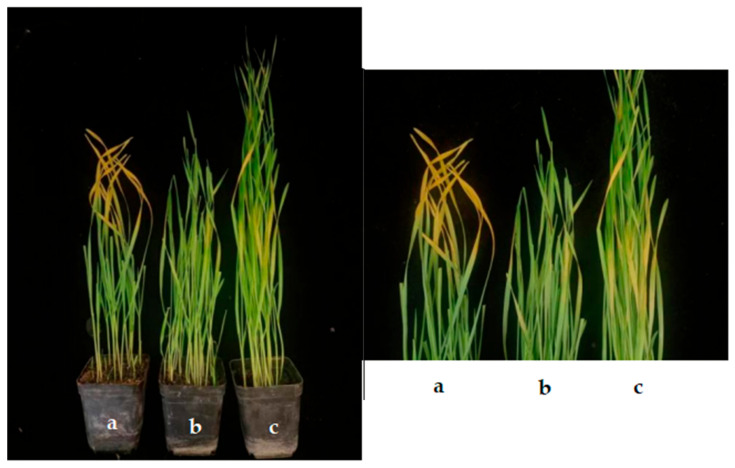
Biological control effect of antagonistic strains on wheat stripe rust; (**a**) water control; (**b**) CS; (**c**) CSS.

The culture solution (CS) of XD29-Y1, XD29-Y2, XD5-J1, XD14-J3, and XD41-J8 showed higher inhibition of urediniospore germination than the centrifuged supernatant of the culture solution (CSS). However, the CSS of XD5-Y2, XD5-G7, XD29-G1, and XD22-G3 showed higher inhibition of urediniospores than its CS. As a whole, the CS of 72 strains showed higher inhibition of urediniospores than its CSS (Figure 1, Figure 2, Figure 3 and Figure 4).

#### 2.3.2. Effect on Control of Wheat Stripe Rust in Pot Experiment

In this study, nine antagonistic endophytic bacteria were used to control wheat stripe rust through a curative effect and a protective effect. The effect of different treatments on wheat stripe rust was different (Table 4, Figure 4). The control efficacy in 24 hbi (protective effect) was significantly higher than in 24 hai (curative effect). The CS of the XD29-G1 strain sprayed in 24 hbi had an obvious control effect on wheat stripe rust, and the control effect was 65.84%. Strain XD5-J1 followed with 60% control efficiency. In 24 hai, the CS and CSS with strain XD29-G1 had the best control effect, with control efficiencies of 62.66% and 60.76%, respectively. Therefore, the most effective antagonist of strain XD29-G1 against wheat stripe rust was screened by pot experiment.

### 2.4. Identification of Antagonistic Strain XD29-G1

#### 2.4.1. Morphological Characteristics

**Figure 5:**
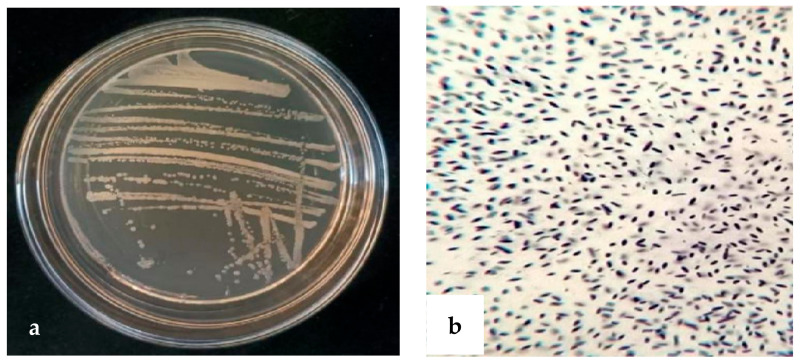
Morphology of strain XD29-G1. (**a**) Bacterial colony morphology; (**b**) Gram reaction microscope.

Antagonistic bacteria strain XD29-G1 was cultured on an NA agar plate at 28 °C for 24 h, and we observed the morphological characteristics. The colony was round, opaque, milky white, and sticky. The Gram reaction was positive (Figure 5).

#### 2.4.2. Biochemical and Physiological Traits

Biochemical and physiological tests were performed on the antagonist XD29-G1 according to the standard methods in the “Identification System Manual of Common Bacteria”. Biochemical tests indicated that the strain was able to use the catalase activity, starch hydrolyses activity, gelatin liquefaction, nitrate reduction activity, and V-P test. The indole test, methyl red activity, citrate activity, propionate, and amino acid decarboxylase test were negative. The D-xylose, glucose activity, maltose activity, and L-Arabinose tests were able to be used, but the D-mannitol activity was unavailable. At 7% sodium oxide and pH 5.7, it could not grow (Table 5).

#### 2.4.3. Molecular Biological Characteristics

**Figure 6:**
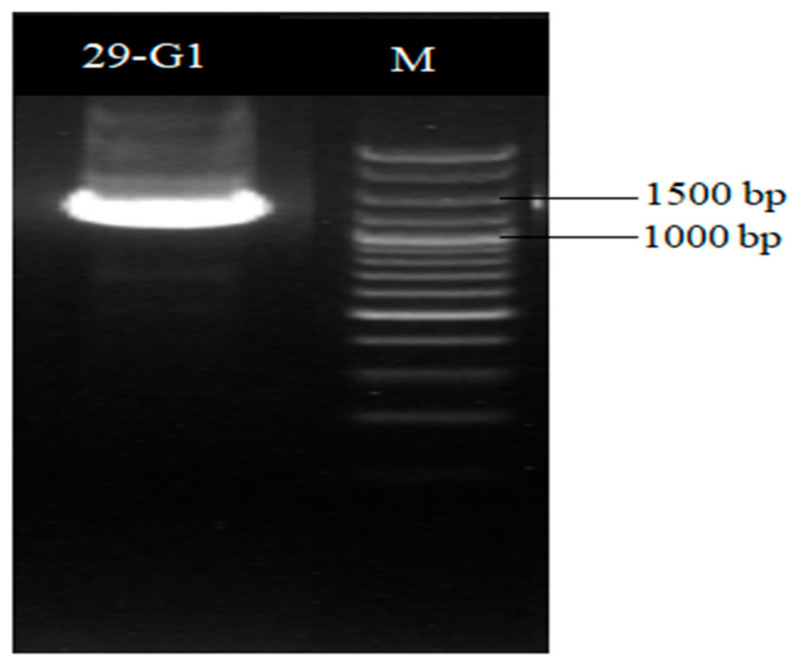
16S rDNA PCR amplification of antagonist XD29-G1.

**Figure 7:**
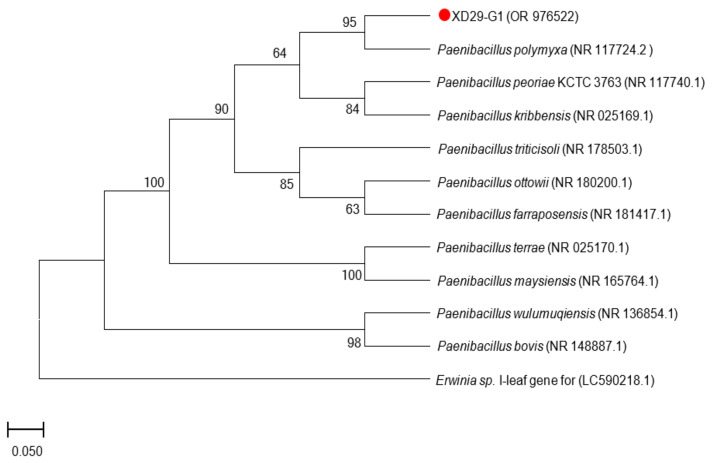
Phylogenetic tree of strain XD29-G1 based on 16S rDNA sequence.

A 16S rDNA sequence and a homology analysis of strain XD29-G1 were performed, and we constructed the phylogenetic tree using MEGA software (version 7.0, Mega Limited, Auckland, New Zealand). The 16S rDNA sequence length of strain XD29-G1 was 1490 bp (Figure 6). As shown in Figure 7, *Paenibacillus polymyxa* (NR 117724.2) was the most similar strain to strain XD29-G1, with a similarity of 99.66%. These results confirm that strain XD29-G1 is an isolate of *Paenibacillus* sp. (GenBank accession number OR976522).

**Figure 8:**
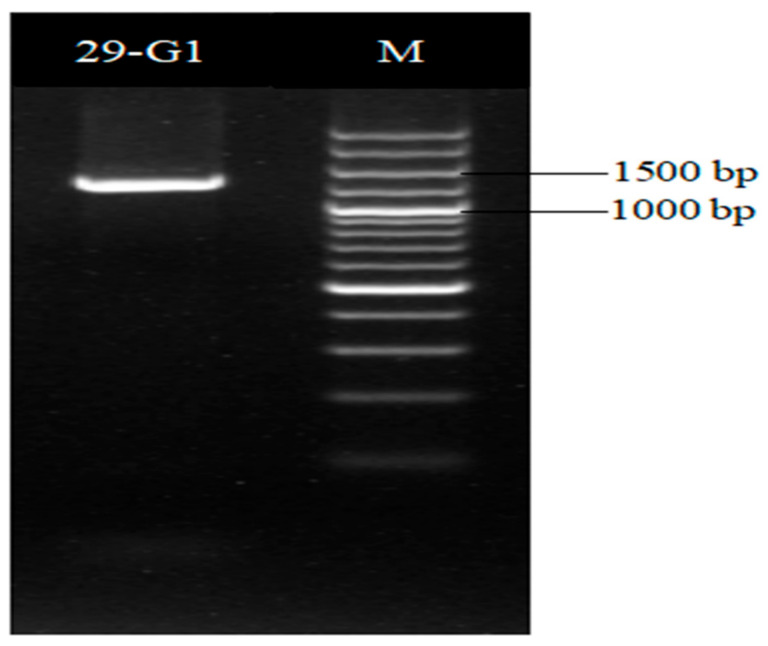
*gyrB* PCR amplification of antagonist XD29-G1.

**Figure 9:**
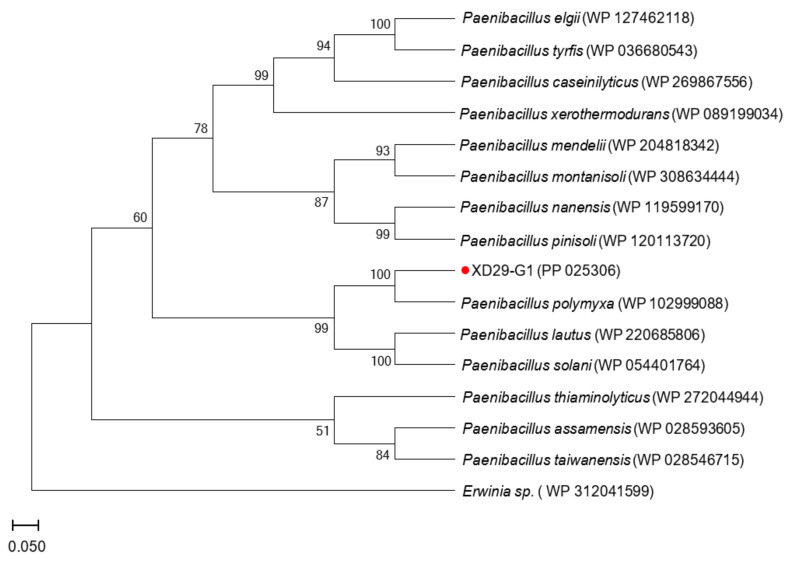
Phylogenetic tree of strain XD29-G1 based on *gyrB* gene sequence.

The *gyrB* gene sequence length of strain XD29-G1 was 1151 bp (Figure 8), and the BLAST analysis showed that it had the highest homology with *Paenibacillus polymyxa* (WP 102999088). The phylogenetic tree analysis showed that strain XD29-G1 was closely related to *Paenibacillus polymyxa* and clustered on one branch (Figure 9). Therefore, through morphological, biochemical, and molecular biological characteristics, the preliminary identification of strain XD29-G1 is *Paenibacillus polymyxa*.

### 2.5. Effect of XD29-G1 on Wheat Seed Germination

**Figure 10:**
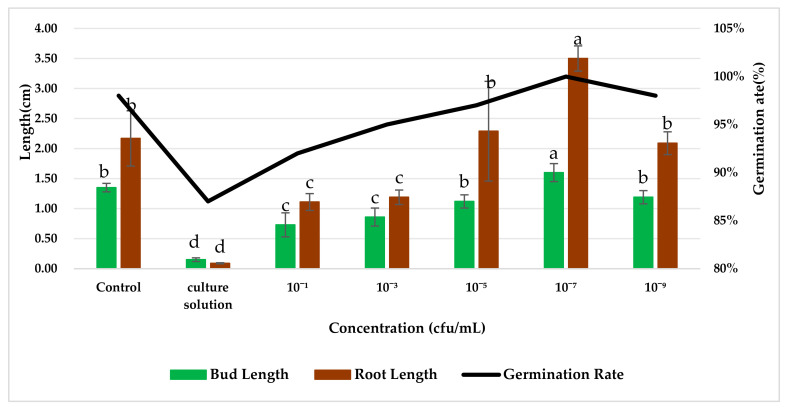
Effects of strain XD29-G1 on wheat seed germination. Values in charts are mean ± standard deviation. Different letters indicate significant differences at the *p* < 0.005 level after Duncan’s test.

**Figure 11:**
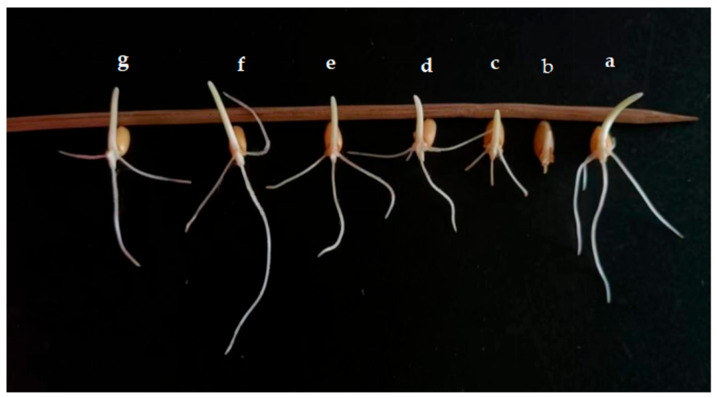
Effects of different dilution concentrations on wheat seed germination. (**a**) Control; (**b**) culture solution; (**c**) 10^−1^ cfu/mL; (**d**) 10^−3^ cfu/mL; (**e**) 10^−5^ cfu/mL; (**f**) 10^−7^ cfu/mL; (**g**) 10^−9^ cfu/mL.

The effect of the application of endophytic bacteria XD29-G1 on wheat seed germination is presented in Figure 10 and Figure 11. The germination rate, bud length, and root length of wheat seed treated with XD29-G1 culture solution (CS) were the lowest at 86.67%, 0.15 cm, and 0.09 cm, respectively. XD29-G1 at the concentration of 10^−7^ cfu/mL demonstrated the greatest effect on the promotion of wheat seed germination compared to other concentrations. The germination rate, bud length, and root length were 100%, 1.60, and 3.50 cm, respectively The results indicated that when the concentration of XD29-G1 was 10^−7^ cfu/mL, the promotion effect was most obvious.

### 2.6. Effect of XD29-G1 on Growth of Wheat at Seedling Stage

**Figure 12:**
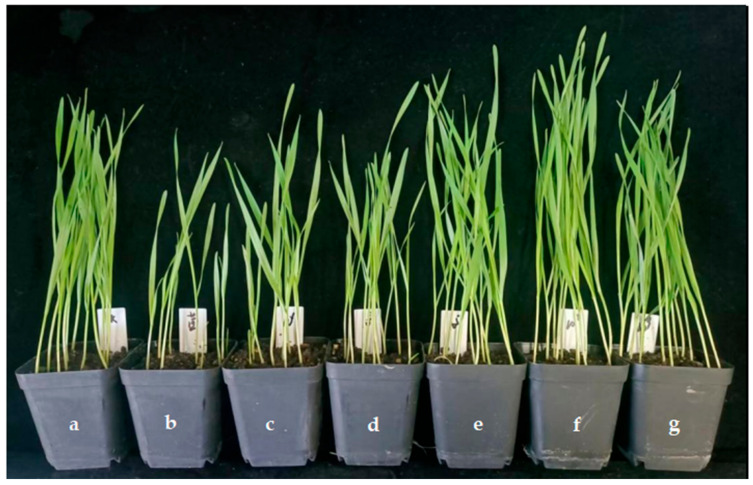
Effects of XD29-G1 on the growth of wheat at the seedling stage. (**a**) Control; (**b**) culture solution; (**c**) 10^−1^ cfu/mL; (**d**) 10^−3^ cfu/mL; (**e**) 10^−5^ cfu/mL; (**f**) 10^−7^ cfu/mL; (**g**) 10^−9^ cfu/mL.

The effect of the application of endophytic bacteria XD29-G1 on the growth of wheat seedlings is presented in Table 6 and Figure 12. The plant height, fresh weight, and dry weight of the wheat seedlings treated with the culture solution (CS) were the lowest at 34.73 cm, 0.090 cm, and 0.024 g, respectively. When the concentration was 10^−5^ cfu/mL, 10^−7^ cfu/mL, and 10^−9^ cfu/mL, the growth index of wheat seedlings was higher than the control group. The concentration of 10^−7^ cfu/mL promoted the plant height, fresh weight, and dry weight of the wheat seedlings, which were 46.33 cm, 0.348 cm, and 0.041 g, respectively. It can be seen that the high concentration inhibited the growth of wheat seedlings, while the low concentration promoted the growth of wheat seedlings. The optimal concentration for the growth of wheat seedlings was 10^−7^ cfu/mL.

### 2.7. Antagonistic Effect of XD29-G1 on Wheat Stripe Rust

The antagonistic effect of XD29-G1 on wheat stripe rust is shown in Table 7. Different concentrations of antagonist XD29-G1 suspension showed certain control effects on WSR, but when the concentration of the bacterial solution decreased, the biocontrol effect also showed a decline.

The control effect on wheat stripe rust of the culture solution was 55.97%. The control effect reached 61.19% when the concentration was diluted to 10^−1^ cfu/mL, and there was no significant difference compared to the culture solution with the concentration of 10^−1^ cfu/mL. But, the control efficiency decreased when the concentration of the culture solution decreased.

## 3. Discussion

As a biological control strain, plant endophytic bacteria have many advantages [39] and play a very important role in the prevention and control of diseases caused by fungi and bacteria. In this study, endophytic bacteria were isolated, screened, and identified from different cultivars of winter wheat in Xinjiang. The results showed that among 136 strains of endophytic bacteria isolated from different parts of healthy wheat, 9 strains with antagonistic effects were screened initially, and the inhibition rate was more than 70%. In the deep pot screening test, strain XD29-G1 had the best control effect on wheat stripe rust, and the control effect was more than 60%. Based on morphological characteristics, physiological characteristics, biochemical characteristics, and molecular biological identification, strain XD29-G1 was identified as *Paenibacillus polymyxa*. In the pot experiment, *Paenibacillus polymyxa* XD29-G1 showed certain growth-promotion effects on wheat seed germination and seedling growth, and it had a control effect on WSR. Therefore, in this study, the antagonistic strain XD29-G1 was isolated and screened as a potential biocontrol agent to control wheat stripe rust.

At present, more than 120 species of endophytic bacteria have been found in various crops fruit trees and other cash crops, of which *Pseudomonas*, *Bacillus*, *Microbacterium*, *Paenibacillus*, and *Agrobacterium* are the most common genera [40]. Pang [41] randomly selected 127 strains of wheat endophytic bacteria isolated from 610 strains belonging to 10 genera and 22 species, among which *Bacillus* was the most dominant, followed by *Pseudomonas*. Cun [42] identified 174 strains of endophytic bacteria from maize, which belonged to 25 genera, of which *Bacillus* accounted for 52%, *Streptomyces* accounted for 13%, *Paenibacillus* accounted for 6%, and *Pseudomonas* accounted for 5%. These results indicate that there are a large number of strains of different genera in the plant, of which *Bacillus* and *Pseudomonas* are the dominant populations. In this study, through 16S rDNA sequence analysis, 136 endophytic bacteria were found to belong to 38 genera, among which *Pseudomonas* accounted for 21% and *Microbacterium*, and *Paenibacillus* followed, accounting for 10%, followed by *Bacillus* and *Agrobacterium* at 8%. However, there are differences in the distribution of dominant populations, which may due to different crop types or related to environmental climate conditions.

The *Pst* has five types of spores. The asexual stage produces urediniospores, and the urediniospore stage is the main way to complete the life history. Urediniospores play an important role in disease development and the spread of *Pst*. Therefore, urediniospores play a decisive role in effectively controlling *Pst*. In this study, nine endophytic bacteria with strong inhibition of spore germination of urediniospores were selected in the preliminary screening test. Among them, XD5-J1, XD5-G7, and XD29-G1 strongly inhibited the germination of urediniospores, and the inhibition rate was more than 85%. The research results were consistent with the research results of the strains WCS358::*phl*, CN078, and CN124 and their inhibition of urediniospores [43] and wheat root endophytic *Bacillus subtilis* E1R-j [44]. The nine antagonists were screened in the pot test. The pot test results showed that the nine antagonists had different control effects on wheat stripe rust, and the antagonist XD29-G1 had the best control effect. The antagonist XD29-G1 could significantly reduce the disease index of wheat stripe rust before *Pst* inoculation, but when sprayed after inoculation, it could not significantly reduce the disease index. It may be that the antagonists secrete antibiotic substances to inhibit the germination of urediniospores, thus affecting the disease index of wheat stripe rust. The effect of spraying antagonists after inoculation was not as good as that before inoculation, which may be due to the weak colonization ability of antagonists in wheat leaves or insufficient time to secrete antibiotic substances.

In addition to morphological identification, the identification method for bacteria should be combined with Gram staining, fatty acid determination, physiological and biochemical reactions, and molecular biological means. With the further development of sequencing technology, by measuring the sequence of bacterial-specific gene fragments, a more convenient method is provided for the accurate identification of bacteria. For example, 16S rDNA, *rpoA*, *rpoB*, *gyrA*, *gyrB*, and other genes are used [45]. The variation in the *gyrB* gene sequence can distinguish members of the *Paenibacillus polymyxa* group [46]. In this study, the morphological identification, physiological and biochemical tests, and 16S rDNA of the XD29-G1 strain were determined, and the strain was preliminarily identified as a genus, but the species could not be determined. The *gyrB* gene sequence was further defined, and the strain *Paenibacillus polymyxa* (WP 102999088) was divided into the same strain. The strainXD29-G1 was identified as *Paenibacillus polymyxa*.

The results of growth promotion showed that different dilution concentrations of XD29-G1 had different promoting effects on wheat seed germination and wheat seedling growth. Among them, the optimal promoting concentration for wheat seed germination and wheat seedling growth was 10^−7^ CFU·mL^−1^. However, the growth promotion of endophytic bacteria did not increase with the dilution ratio. Under the condition of an appropriate bacterial dilution concentration, the strains could play a better role in promoting growth, which was confirmed by the studies of Yang [47] and Wang [48]. The greater the dilution ratio of the antagonist XD29-G1, the worse the control effect, which may be due to the lower antagonist bacteria concentration.

*Paenibacillus polymyxa* is a kind of non-pathogenic bacteria to plants, which also has the function of disease prevention and growth promotion and is listed as a class of biocontrol bacteria without safety identification by the Ministry of Agriculture [49]. Some strains of this strain are also important plant biocontrol bacteria and plant rhizosphere growth-promoting bacteria through the production of antibacterial substances or a site competition mode of action, which then induces plants to produce antibacterial substances so as to achieve the purpose of disease control [50,51,52,53]. In this study, the pot test results showed that different dilution concentrations of the strain had different levels of antibacterial activity against WSR, with 10^−1^ CFU·mL^−1^ having the best control effect and a control efficiency of 61.19%. Therefore, *Paenibacillus polymyxa* XD29-G1 had some potential for biocontrol of WSR and serves as a valuable strain resource for the subsequent control of diseases caused by *Pst*. However, the control effect of WSR in the field still needs further exploration.

## 4. Materials and Methods

### 4.1. Plant Material

Endophytic bacteria were isolated from wheat cultivars Xindong No.5, Xindong No.9, Xindong No.14, Xindong No.22, Xindong No.26, Xindong No.29, Xindong No.32, Xindong No.35, and Xindong No.41, cultivated in the greenhouse of Xinjiang Agricultural University, China.

The seeds used in the greenhouse experiments were cultivar Mingxian 169 (highly susceptible to *Pst*) obtained from the Plant Disease Epidemiology Laboratory of Xinjiang Agricultural University, China.

The strain of *Pst* (a mix of popular strains CYR31, CYR32, and CYR33) was acquired from the Laboratory of Plant Disease Epidemiology of Xinjiang Agricultural University and Institute of Plant Protection, Xinjiang Academy of Agricultural Sciences. The *Pst* was used on seedlings after 15 days.

### 4.2. Isolation and Purification of Endophytic Bacteria from Wheat

Endophytic bacteria were isolated through tissue separation and dilution-coated plate methods [54]. Leaf, stem, and root samples were washed with abundant tap water and cut out into small pieces (5 cm long), which were sterilized and treated with 75% ethanol for 1 min and sterile water 3 times, 3% sodium hypochlorite (roots for 6 min, stems for 5 min, leaves for 3 min), and 75% anhydrous ethanol for 30 s, and, finally, cleaned with sterile water. The surface water was blotted with sterile filter paper. The last rinsed sterile water was coated on NA agar for culture as a control, and each treatment was repeated 3 times.

The sterilized samples were macerated by using a sterilized mortar with the addition of sterile distilled water. Different concentrations of bacterial suspensions (10^−3^ to 10^−6^ dilutions) were inoculated on nutrient agar (NA: 1 g of yeast extract, 3 g of beef extract, 10 g of peptone, 10 g of sucrose, 5 g of sodium chloride, and 15 g of agar in 1000 mL of water) and incubated on the NA agar plates at 28 °C for 2–4 days. Each treatment was repeated 3 times. Pure cultures were obtained through re-growth on NA plates. Pure cultures in 50% glycerol were stored at −80 °C.

### 4.3. Identification and Diversity Analysis of Endophytic Bacteria

The genomic DNA of endophytic bacteria was extracted through the column method. Bacterial universal primers were used: 27F-(5′-AGAGTTTGATCCTGGCTCAG-3′) [22] and 1492R-(5′-TACGGCTACCTTGTTACGACTT-3′). PCR was performed in a 25 µL reaction mixture containing 2 µL of template DNA, 21.0 µL of PCR Mix, 1 µL of forward primer, and 1 µL of reverse primer. The amplification procedure was an initial 5 min of denaturation at 96 °C, then 35 cycles of 20 s of denaturation at 96 °C, and then 30 s of annealing at 62 °C. Step extension was conducted for 30 s at 72 °C. The genomic DNA of endophytic bacteria was amplified through PCR, and the primers were synthesized by Beijing BGI Biotechnology Co., Ltd. (Beijing, China). BLAST from GenBank was applied to compare the endophytic bacteria homology.

In order to analyze the diversity of endophytic bacteria among different wheat varieties, the analysis method for endophytic bacteria diversity was calculated according to Liu [55]. The Simpson diversity index (D) and the Shannon diversity index (H) were calculated to evaluate the diversity of wheat endophytic bacteria. The calculation formula is as follows ((1) and (2)):

Simpson diversity index (D):

(1) $$D=1-\sum_{i}^{s}\frac{n_{i}\left(n_{i}-1\right)}{N\left(N-1\right)}$$

Shannon diversity index (H):

(2) $$H=-\sum_{i=1}^{s}p_{i}\ln p_{i}$$

where *p_i_* is the ratio of species *i* to the total number of strains isolated, *S* is the number of endophytic bacteria species isolated from each site, *n_i_* is the number of strains of species *i*, and *N* is the total number of strains isolated from each tissue.

### 4.4. Screening of Antagonistic Endophytic Bacteria

#### 4.4.1. Inhibitory Effect of Endophytic Bacteria on the Germination of Urediniospores

The strongly antagonistic endophytic bacteria were screened through the water agar plate method [21]. Cultures of the strains were obtained by transferring individual colonies to 250 mL conical flasks and culturing them at 28 °C with shaking at 180 rpm for 24 h (OD_600_ = 1). The culture solution (CS) and centrifuged supernatant of the culture solution (CSS) were prepared. The CS was centrifuged at 10,000× *g* for 20 min at 4 °C, and the supernatant was collected as the centrifuged supernatant of the culture solution.

The water agar was melted and, using a pipette gun, 1 mL was placed on a slide to make a water agar film [56], which was placed in a Petri dish with wet filter paper. Then, 200 µL of CS and CSS was coated onto water agar slides, and the urediniospores were uniformly sprinkled on the liquid surface. A total of 150 spores were used in each treatment. Agar plates without bacterial culture were used as controls. The germinated urediniospores on water agar were observed under a microscope after incubation at 9 °C for 12 h, as well as the growth and germination of urediniospores. Spore germination was counted when the length of the germ tube reached half the diameter of the urediniospores [57]. The experimental designs were completely randomized, with 3 replicates for each treatment, and repeated 3 times. The strain with the best inhibitory effect on the germination of urediniospores was selected for subsequent experiments. The calculation formula is as follows ((3) and (4)):

(3) $$\mathrm{Germination}\;\mathrm{Rate}\;(\%)=\frac{\mathrm{number}\;\mathrm{of}\;\mathrm{spores}\;\mathrm{germinated}}{\mathrm{total}\;\mathrm{number}\;\mathrm{of}\;\mathrm{spores}\;\mathrm{investigated}}\times 100$$

(4) $$\mathrm{Germination}\;\mathrm{Inhibition}\;\mathrm{Rate}\;(\%)=\frac{\mathrm{control}\;\mathrm{spore}\;\mathrm{germination}\;\mathrm{rate}-\mathrm{treatment}\;\mathrm{spore}\;\mathrm{germination}\;\mathrm{rate}}{\mathrm{control}\;\mathrm{spore}\;\mathrm{germination}\;\mathrm{rate}}\times 100$$

#### 4.4.2. Effect on Control of Wheat Stripe Rust in Deep Pot Test

Deep pot tests were conducted to evaluate the control effects of the selected strains on *Pst* [21]. The susceptible wheat cultivar “Ming Xian 169” was sown in pots. Seven-day-old seedlings were used to determine the control effects of the selected strains based on their protective and curative effects. To evaluate the curative effect, seedlings were sprayed with CS and CSS 24 h after inoculation with urediniospores. To evaluate the protective effect, wheat seedlings were sprayed 24 h before urediniospore inoculation. The urediniospores of wheat stripe rust were prepared in 20 mg/mL of spore suspension with electron fluoridation solution, mixed evenly, and inoculated with 2.5 µL of spore suspension. The inoculated wheat seedlings were maintained at 10 °C for 24 h in the dark and then transferred to a climate chamber with a 16/8 h cycle (light/dark cycle) that had been set at 11–13 °C (light/dark period). Control seedlings were sprayed with sterile water. Three pots each with 10 wheat seedlings were used for each treatment. After 14 d of incubation, when the control plants reached the maximum sporulation, the incidence of plant leaves was investigated, and the disease index (DI) and control effects (CE) were calculated as follows ((5) and (6)):

(5) $$DI=\frac{\sum_{i=0}^{n}\left(X_{i}\times S_{i}\right)}{\sum_{i=0}^{n}\left(X\times S_{\max}\right)}\times 100$$

(6) $$CE_{treatment}\left(\%\right)=\frac{DI_{control}-DI_{treatment}}{DI_{control}}\times 100$$

**Figure 13:**
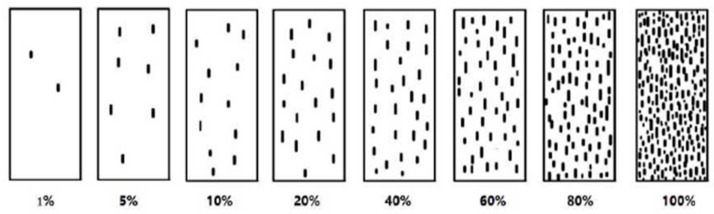
Classification criteria for wheat stripe rust severity.

Severity was classified from 1 to 8 levels corresponding to a coverage of 1, 5, 10, 20, 40, 60, 80, and 100% according to the percentage of leaf areas covered by the urediniospores [58] (Figure 13). *i* indicates the disease grade, *X_i_* consists of the number of leaves with disease grade *i*, *S_i_* is the value of the disease grade *I*, and *X* represents the total number of investigated leaves, while *S_max_* shows the highest severity grade. Furthermore, *treatment* indicates different treatments, *control* indicates blank control, and *CE_treatment_* is the control efficiency of different treatments (these classifications are defined by NY/T 1443.2-2007 [59], the agricultural industry standard of the People’s Republic of China).

### 4.5. Identification of Antagonistic Strain XD29-G1

#### 4.5.1. Morphological Characteristics and Biochemical and Physiological Traits

The antagonistic bacteria were used in the inoculation in NA agar, incubated at 28 °C for 24 h, and then characterized through a Gram reaction. The color, size, shape, transparency, edge uniformity, and other morphological characteristics of the colonies were observed. The biochemical and physiological traits of the antagonists were identified according to the method in the “Identification System Manual of Common Bacteria” [60].

#### 4.5.2. Molecular Biological Characteristics

The molecular biological characteristics of antagonistic bacteria were identified using 16S rDNA and the *gyrB* gene. *GyrB* gene primers were selected for amplification and sequencing verification [61]. The PCR amplification method of 16S rDNA is described in the identification and diversity analysis of endophytic bacteria. The *gyrB* gene sequence was amplified through PCR using *gyrB* gene primers UP2F-(5′-AGCAGGGTACGGATGTGCGAGCCRTCNACRTCNGCRTCNGTCAT-3′) and UP2R-(5′-GAAGTCATCATGACCGTTCTGCAYGCNGGNGGNAARTTYGA-3′). The genomic DNA of endophytic bacteria was amplified through PCR, and the primers were synthesized by Shenggong Bioengineering (Shanghai) Co., Ltd. (Shanghai, China). The 16S rDNA and *gyrB* sequences obtained through sequencing were blasted on NCBI, and the gene sequences of model strains with high homology were used as reference objects. Mega software (version 7.0, Mega Limited, Auckland, New Zealand) was used to construct the phylogenetic tree to determine the taxonomic status of antagonistic strains.

### 4.6. Effect of Antagonistic Endophytic Bacteria on Wheat Seed Germination

The wheat seeds were washed with tap water to remove debris on the surface, disinfected with 70% alcohol for 3 min, soaked in 1% sodium hypochlorite solution for 10 min, and then washed with sterile water 5 times [62]. The seeds were inoculated with the antagonistic endophytic bacteria (XD29-G1) and 20 mL of NB culture medium, centrifuged at 28 °C for 180 r/min, and then diluted with sterilized water to create bacterial suspensions with concentrations of culture solution (CS) of 10^−1^ CFU·mL^−1^, 10^−3^ CFU·mL^−1^, 10^−5^ CFU·mL^−1^, 10^−7^ CFU·mL^−1^, and 10^−9^ CFU·mL^−1^.

Next, 5 mL of each dilution of XD29-G1 was used to add to a Petri dish containing 20 wheat seeds as the inoculation. Sterile water was used as the control. There were 20 wheat seeds in each Petri dish, and the test was repeated 3 times. All seeds were incubated at 28 °C in the incubator. When the first seed germinated (the bud was longer than half the length of the seed and the root length was equal to the length of the seed as the standard), the germination rate, bud length, and root length were measured and recorded. The seed germination rate was calculated as follows (7):

(7) $$\mathrm{Germination}\;\mathrm{rate}\;(\%)=(\frac{\mathrm{Total}\;\mathrm{germination}\;\mathrm{rate}\;\mathrm{of}\;\mathrm{tested}\;\mathrm{seeds}}{\mathrm{Total}\;\mathrm{number}\;\mathrm{of}\;\mathrm{seeds}\;\mathrm{tested}})\times 100$$

### 4.7. Effect of Antagonistic Endophytic Bacteria on Growth of Wheat at Seedling Stage

Wheat seeds were surface-disinfected and kept in an incubator at 28 °C for 24 h in the dark to promote germination. After that, the wheat seeds were treated with five different concentrations of XD29-G1 suspensions and sown in pots of 12–15 seeds per pot with three replications per treatment and incubated in the greenhouse. On the 15th day after seed sowing, the soil was rinsed from the roots of the plants with distilled water. The plant height and fresh and dry weights of the wheat plants were measured.

### 4.8. Antagonistic Effect of Endophytic Bacteria on Wheat Stripe Rust

Wheat seeds were surface-disinfected and treated with five different concentrations of XD29-G1 suspensions. Sterile water was used as the control. The seeds were incubated in an incubator at 28 °C for 48 h for germination. Subsequently, wheat seeds were then seeded in pots of 10–12 seeds each. After 15 days, wheat seedlings were treated by spraying different concentrations of XD29-G1 suspensions; then, 24 h later, the wheat was inoculated with *Pst*. Mixing the urediniospores of *Pst* with electron-fluorinated solution produced a 20 mg/mL spore suspension, and then the wheat seedlings were inoculated with the spore suspension using 2.5 µL. The inoculated seedlings were maintained at 10 °C for 24 h in the dark and then transferred to an artificial climate chamber with a 16/8 h cycle (light/dark cycle) set at 11 °C. When the control plants reached the maximum sporulation ratio, the disease index of the plant was investigated, and the disease statistics were carried out according to the 8-grade criteria (Figure 13).

### 4.9. Statistical Analysis

The number of endophytic bacteria and the species diversity data were statistically analyzed using Microsoft Excel 2019 software. All results are the means of three independent replicates. Data are presented as means ± standard deviations (S.D.). The data regarding the urediniospore germination rate, the disease index, the control effect of different treatments, the bud growth, the root length, the plant height, the fresh weight, and the dry weight were subjected to analysis of variance (ANOVA) followed by Duncan’s multiple range tests (*p* ≤ 0.005) using SPSS statistical software (version 26.0, IBM Corporation, New York, NY, USA). MEGA software (version 7.0, Mega Limited, Auckland, New Zealand) was applied for sequence comparison and cluster analysis, and the Neighbor-Joining method was applied to construct the phylogenetic tree.

## 5. Conclusions

A total of 136 strains of endophytic bacteria were isolated from different tissues of healthy winter wheat, among which 9 strains significantly inhibited the germination of urediniospores. During the deep pot test, strain XD29-G1 was effective against stripe rust in wheat. The strain XD29-G1 was identified as *Paenibacillus polymyxa*. According to the growth promotion test, *Paenibacillus polymyxa* XD29-G1 had a certain promotion effect on wheat seed germination and seedling growth. The best concentration of growth-promoting dilution was 10^−7^ CFU·mL^−1^. The results of the pot test to control WSR showed that XD29-G1 had an excellent control effect at 61.19%. In this study, a strong antagonist strain was obtained for control *Pst*, which provided strain resources for WSR biological control.

## Figures and Tables

**Table 1:** The number of endophytic bacteria in different tissues of different wheat cultivars.

Wheat Cultivars	Leaf	Stem	Root	Total
Xindong No.5	5	2	8	15
Xindong No.9	2	8	4	14
Xindong No.14	0	3	8	11
Xindong No.22	0	5	4	9
Xindong No.26	2	4	8	14
Xindong No.29	2	8	7	17
Xindong No.32	1	7	11	19
Xindong No.35	0	8	4	12
Xindong No.41	3	9	13	25
Total	15	54	67	136

**Table 2:** Species diversity of endophytic bacteria in different tissues of wheat.

Classification	Number of Endophytic Bacteria			
	Leaf	Stem	Root	Sum
Total number of isolates (N)	15	54	67	136
Species richness (S)	10	23	24	38
Shannon diversity index (H)	2.079	2.710	2.697	3.214
Simpson diversity index (D)	0.896	0.915	0.913	0.921

**Table 3:** Species diversity of endophytic bacteria in different wheat cultivars.

Wheat Cultivars	Total Number of Isolates (N)	Species Richness (S)	Shannon Diversity Index (H)	Simpson Diversity Index (D)
Xindong No.5	15	8	1.876	0.933
Xindong No.9	14	10	2.168	0.934
Xindong No.14	11	7	1.846	0.909
Xindong No.22	9	6	1.677	0.889
Xindong No.26	14	12	2.441	0.978
Xindong No.29	17	9	1.972	0.882
Xindong No.32	19	10	2.032	0.895
Xindong No.35	12	8	1.979	0.924
Xindong No.41	25	18	2.754	0.963
Total	136	38	2.965	0.921

**Table 4:** Control effect of antagonistic strains on wheat stripe rust.

Endophytic Bacteria	Protective Effect (24 hbi)					Curative Effect (24 hai)				
	Control	CS		CSS		Control	CS		CSS	
	Disease Index (%)	Disease Index (%)	Control Efficiency (%)	Disease Index (%)	Control Efficiency (%)	Disease Index (%)	Disease Index (%)	Control Efficiency (%)	Disease Index (%)	Control Efficiency (%)
XD5-Y2	42.93 ± 2.57 a	27.20 ± 2.12 b	36.65	23.47 ± 5.33 b	45.34	42.13 ± 8.40 a	19.47 ± 3.33 b	53.80	23.73 ± 7.26 bc	43.67
XD29-Y1		19.47 ± 4.69 bc	54.66	21.60 ± 0.80 b	49.69		17.33 ± 5.90 b	58.86	18.93 ± 6.42 bc	55.66
XD29-Y2		18.13 ± 3.23 bc	57.76	22.40 ± 5.54 b	47.83		16.80 ± 1.39 b	60.13	26.40 ± 2.77 b	37.34
XD5-J1		17.33 ± 3.33 bc	59.63	16.80 ± 4.80 b	60.87		23.47 ± 3.78 b	44.30	17.60 ± 6.40 c	58.23
XD14-J3		21.06 ± 1.85 bc	50.93	22.93 ± 4.41 b	46.58		22.93 ± 1.67 b	45.57	24.27 ± 3.33 bc	42.41
XD41-J8		23.20 ± 10.59 bc	45.96	19.47 ± 3.23 b	54.66		21.07 ± 5.33 b	50.00	18.67 ± 4.40 bc	55.70
XD5-G7		21.07 ± 9.20 bc	50.93	21.87 ± 3.23 b	49.07		22.40 ± 8.91 b	46.84	17.87 ± 6.00 bc	57.59
XD22-G3		18.40 ± 4.90 bc	57.14	24.27 ± 9.54 b	43.48		24.00 ± 7.63 b	43.67	20.00 ± 2.12 bc	56.33
XD29-G1		14.67 ± 5.14 c	65.84	17.60 ± 2.57 b	60.25		16.27 ± 5.79 b	62.66	16.53 ± 5.79 bc	60.76

Note: XD is Xindong; Y is the leaf; J is the stem; G is the root; CS is the culture solution; CSS is the centrifuged supernatant of the culture solution. Data presented here are mean ± standard deviation, and different letters in the same column indicate significant differences at the *p* < 0.005 level after Duncan’s method test.

**Table 5:** Biochemical and physiological traits of antifungal strain XD29-G1.

Biochemical and Physiological Traits	XD29-G1	Biochemical and Physiological Traits	XD29-G1
Catalase Activity	+	D-Xylose	+
Glucose Activity	+	L-Arabinose	+
Maltose Activity	+	D-mannitol Activity	-
Indoletest	-	Gelaune Liquefaction	+
Methyl Red Activity	-	7% Sodium Oxide Growth	-
Amino Acid Decarboxylase	-	pH 5.7 Growth	-
V-P Test	+	Nitrate Reduction Activity	+
Citrate Activity	-	Starch Hydrolyses Activity	+
Propionate	-		

Note: “+” indicates positive reaction and “-” indicates negative reaction.

**Table 6:** Effects of XD29-G1 on wheat growth at seedling stage.

Concentration (cfu/mL)	Plant Height (cm)	Fresh Weight (Plant/g)	Dry Weight (Plant/g)
Control	42.67 ± 1.76 ab	0.297 ± 0.039 a	0.036 ± 0.003 ab
Culture solution	34.73 ± 3.10 c	0.090 ± 0.009 c	0.024 ± 0.008 c
10^−1^	40.13 ± 1.00 b	0.302 ± 0.040 a	0.036 ± 0.001 ab
10^−3^	40.07 ± 0.40 b	0.226 ± 0.019 b	0.034 ± 0.004 b
10^−5^	44.17 ± 1.04 a	0.306 ± 0.034 a	0.037 ± 0.001 ab
10^−7^	46.33 ± 3.55 a	0.348 ± 0.035 a	0.041 ± 0.002 a
10^−9^	45.33 ± 1.60 a	0.332 ± 0.088 a	0.040 ± 0.002 ab

Note: The data in the table are mean ± standard deviation, and different letters in the same column indicate significant differences at the *p* < 0.005 level after Duncan’s test.

**Table 7:** Antagonistic effect of XD29-G1 on wheat stripe rust.

Concentration (cfu/mL)	Disease Index	Control Efficiency (%)
Control	35.73 ± 3.70 a	-
Culture solution	15.70 ± 4.11 bc	55.97
10^−1^	13.87 ± 2.31 c	61.19
10^−3^	19.73 ± 12.93 bc	44.78
10^−5^	20.80 ± 4.45 bc	41.79
10^−7^	24.53 ± 1.67 bc	31.34
10^−9^	25.87 ± 5.21 ab	27.61

Note: The data in the table are mean ± standard deviation, and different letters in the same column indicate significant difference at the *p* > 0.005 level after Duncan’s method test.

## Data Availability

Data are contained within the article.

## Appendix A

**Table A1:** Identification results of endophytic bacteria in wheat.

Strain Number (Accession Number)	Closest Similar Species (Accession Number)	Strain Number (Accession Number)	Closest Similar Species (Accession Number)
XD5-Y1 (OP591349)	98.83% to *Microbacterium thalassium* (NR04248.1)	XD29-J7 (OP594419)	99.49% to *Pseudomonas kilonensis* (NR028929.1)
XD5-Y2 (OP591350)	99.85% to *Microbacterium oryzae* (NR117527.1)	XD29-J8 (OP594420)	100.00% to *Pseudomonas plecoglossicida* (NR024662.1)
XD5-Y3 (OP591351)	99.34% to *Microbacterium schleiferi* (NR044936.1)	XD29-J9 (OP594421)	98.30% to *Salmonella enterica* (NR074910.1)
XD5-Y4 (OP591352)	99.65% to *Stenotrophomonas maltophilia* (NR041577.1)	XD29-J10 (OP594422)	99.49% to *Pseudomonas kilonensis* (NR028929.1)
XD5-Y5 (OP591353)	99.45% to *Massilia timonae* (NR026014.1)	XD29-J11 (OP594423)	99.32% to *Bacillus tropicus* (NR157736.1)
XD5-J1 (OP591347)	99.86% to *Staphylococcus hominis* (NR041323.1)	XD29-G1 (OR976522)	999.73% to *Paenibacillus polymyxa* (NR114810.1)
XD5-J2 (OP591348)	99.43% to *Kocuria polaris* (NR028924.1)	XD29-G2 (OP594410)	99.58% to *Pseudomonas brassicacearum* (NR116299.1)
XD5-G2 (OP591339)	99.20% to *Pseudomonas kilonensis* (NR028929.1)	XD29-G4 (OP594411)	99.31% to *Pseudomonas kilonensis* (NR028929.1)
XD5-G3 (OP591340)	100.00% to *Pseudomonas brassicacearum* (MT634582.1)	XD29-G5 (OP594412)	99.86% to *Bacillus tropicus* (NR157736.1)
XD5-G5 (OP591341)	99.79% to *Microbacterium oryzae* (NR117527.1)	XD29-G6 (OP594413)	99.72% to *Paenibacillus polymyxa* (NR114810.1)
XD5-G6 (OP591342)	99.93% to *Pseudomonas plecoglossicida*(NR024662.1)	XD29-G7 (OP594414)	99.86% to *Bacillus velezensis* (NR075005.2)
XD5-G7 (OP591343)	99.65% to *Stenotrophomonas maltophilia* (NR041577.1)	XD29-G8 (OP594415)	99.49% to *Enterobacter cancerogenus* (NR044977.1)
XD5-G9 (OP591344)	99.71% to *Priestia megaterium* (NR112636.1)	XD32-Y3 (OP594444)	99.85% to *Arthrobacter humicola* (NR041546.1)
XD5-G10 (OP591345)	100.00% to *Pseudomonas plecoglossicida* (NR024662.1)	XD32-J1 (OP594437)	99.49% to *Pseudomonas kilonensis* (NR028929.1)
XD5-G11 (OP591346)	100.00% to *Bacillus tropicus* (NR157736.1)	XD32-J2 (OP594438)	99.93% to *Achromobacter spanius* (NR025686.1)
XD9-Y1 (OP591366)	99.28% to *Paenibacillus polymyxa* (NR117732.2)	XD32-J3 (OP594439)	99.79% to *Agrobacterium tumefaciens* (NR116306.1)
XD9-Y2 (OP591367)	98.68% to *Staphylococcus warneri* (NR025922.1)	XD32-J4 (OP594440)	99.79% to *Sphingobium yanoikuyae* (NR113730.1)
XD9-J1 (OP591358)	99.32% to *Enterobacter ludwigii* (NR042349.1)	XD32-J5 (OP594441)	99.70% to *Agrobacterium tumefaciens* (NR116306.1)
XD9-J2 (OP591359)	99.93% to *Agrobacterium tumefaciens* (NR116306.1)	XD32-J6 (OP594442)	99.37% to *Pseudomonas kilonensis* (NR028929.1)
XD9-J4 (OP591360)	98.92% to *Stenotrophomonas maltophilia* (NR041577.1)	XD32-J8 (OP594443)	99.38% to *Fictibacillus halophilus* (NR149289.1)
XD9-J5 (OP591361)	99.93% to *Bacillus atrophaeus* (NR024689.1)	XD32-G3 (OP594426)	99.93% to *Lactococcus lactis* (NR113960.1)
XD9-J6 (OP591362)	99.45% to *Pseudomonas kilonensis* (NR028929.1)	XD32-G4 (OP594427)	98.80% to *Agrobacterium fabacearum* (NR174322.1)
XD9-J7 (OP591363)	99.49% to *Pseudomonas kilonensis* (NR028929.1)	XD32-G5 (OP594428)	99.56% to *Pseudomonas brassicacearum* (NR116299.1)
XD9-J8 (OP591364)	99.31% to *Pseudomonas kilonensis* (NR028929.1)	XD32-G6 (OP594429)	99.58% to *Pseudomonas kilonensis* (NR028929.1)
XD9-J9 (OP591365)	98.09% to *Shinella zoogloeoides* (NR119062.1)	XD32-G8 (OP594430)	99.29% to *Paenibacillus polymyxa* (NR117732.2)
XD9-G1 (OP591354)	99.86% to *Paenibacillus peoriae* (CP092831.1)	XD32-G9 (OP594431)	99.29% to *Paenibacillus polymyxa* (NR117732.2)
XD9-G2 (OP591355)	98.76% to *Microbacterium profundi* (N-044321.1)	XD32-G10 (OP594432)	99.29% to *Paenibacillus polymyxa* (NR117732.2)
XD9-G4 (OP591356)	99.48% to *Paenibacillus peoriae* (NR117743.1)	XD32-G11 (OP594433)	99.93% to *Variovorax boronicumulans* (NR114214.1)
XD9-G5 (OP591357)	98.48% to *Microbulbifer pacificus* (NR115928.1)	XD32-G12 (OP594434)	99.65% to *Pseudomonas kilonensis* (NR028929.1)
XD14-J3 (OP591376)	99.57% to *Pantoea dispersa* (NR116797.1)	XD32-G14 (OP594435)	99.64% to *Streptomyces bellus* (NR041222.1)
XD14-J4 (OP591377)	99.12% to *Peribacillus asahii* (NR024817.1)	XD32-G15 (OP594436)	99.65% to *Pseudomonas kilonensis* (NR028929.1)
XD14-J5 (OP591378)	99.72% to *Agrobacterium larrymoorei* (NR026519.1)	XD35-J1 (OP598852)	99.51% to *Pseudomonas kilonensis* (NR028929.1)
XD14-G1 (OP591368)	99.41% to *Thalassospira xianhensis* (NR116127.1)	XD35-J2 (OP598851)	100.00% to *Agrobacterium tumefaciens* (NR041396.1)
XD14-G2 (OP591369)	99.64% to *Stenotrophomonas maltophilia* (NR041577.1)	XD35-J3 (OP622843)	99.14% to *Pantoea agglomerans* (NR114111.1)
XD14-G3 (OP591370)	99.56% to *Thalassospira xianhensis* (NR116127.1)	XD35-J4 (OP597544)	99.20% to *Aurantiacibacter suaedae* (NR169479.1)
XD14-G4 (OP591371)	99.49% to *Pseudomonas kilonensis* (NR02892.1)	XD35-J5 (OP598853)	99.93% to *Microbacterium arborescens* (NR029265.1)
XD14-G5 (OP591372)	99.49% to *Pseudomonas kilonensis* (NR02892.1)	XD35-J7 (OP598854)	98.59% to *Salmonella enterica* (NR074910.1)
XD14-G6 (OP591373)	99.43% to *Paenibacillus polymyxa* (NR112641.1)	XD35-J8 (OP598855)	99.78% to *Pseudomonas punonensis* (NR109583.1)
XD14-G7 (OP591374)	99.37% to *Thalassospira xianhensis* (NR116127.1)	XD35-J9 (OP598856)	99.49% to *Kosakonia cowanii* (NR025566.1)
XD14-G8 (OP591375)	99.93% to *Bacillus tropicus* (NR157736.1)	XD35-G1 (OP598857)	100.00% to *Agrobacterium tumefaciens* (CP042275.2)
XD22-J1 (OP591383)	99.86% to *Staphylococcus capitis* (NR036775.1)	XD35-G2 (OP598858)	99.57% to *Kocuria polaris* (NR028924.1)
XD22-J2 (OP591384)	99.70% to *Stenotrophomonas pavanii* (NR118008.1)	XD35-G3 (OP598859)	99.64% to *Pseudomonas kilonensis* (NR028929.1)
XD22-J3 (OP591385)	99.93% to *Agrobacterium fabacearum* (NR174322.1)	XD35-G4 (OP598860)	98.84% to *Microbacterium thalassium* (NR042481.1)
XD22-J4 (OP591386)	99.93% to *Bacillus velezensis* (NR075005.2)	XD41-Y1 (OP597547)	99.63% to *Microbacterium foliorum* (NR025368.1)
XD22-J5 (OP591387)	99.59% to *Pseudomonas kilonensis* (NR028929.1)	XD41-Y2 (OP597548)	99.52% to *Pseudomonas kilonensis* (NR028929.1)
XD22-G1 (OP591379)	99.93% to *Bacillus tropicus* (NR157736.1)	XD41-Y3 (OP597549)	99.28% to *Microbacterium saccharophilum* (NR114342.1)
XD22-G2 (OP591380)	99.57% to *Pantoea dispersa* (NR116797.1)	XD41-J1 (OP597642)	99.93% to *Lactococcus lactis* (NR040955.1)
XD22-G3 (OP591381)	99.15% to *Agrobacterium tumefaciens* (NR116306.1)	XD41-J2 (OP597643)	98.54% to *Microbacterium thalassium* (NR042481.1)
XD22-G4 (OP591382)	99.93% to *Bacillus tropicus* (NR157736.1)	XD41-J3 (OP597644)	100.00% to *Pseudomonas plecoglossicida* (NR024662.1)
XD26-Y1 (OP594407)	98.94% to *Sphingomonas hankookensis* (NR116570.1)	XD41-J5 (OP597645)	99.50% to *Sphingomonas sanxanigenens* (NR121736.1)
XD26-Y2 (OP594408)	99.34% to *Janibacter melonis* (NR025805.1)	XD41-J6 (OP598868)	99.64% to *Pantoea dispersa* (NR116797.1)
XD26-J1 (OP594403)	99.38% to *Pseudomonas kilonensis* (NR028929.1)	XD41-J7 (OP598869)	99.64% to *Stenotrophomonas maltophiliae* (NR041577.1)
XD26-J3 (OP594404)	99.93% to *Ciceribacter selenitireducens* (NR044216.1)	XD41-J8 (OP598870)	99.19% to *Paenibacillus polymyxa* (NR114810.1)
XD26-J4 (OP594405)	99.49% to *Microbacterium natoriense* (NR042983.1)	XD41-J9 (OP598871)	100.00% to *Agrobacterium tumefaciens* (NR116306.1)
XD26-J5 (OP594406)	98.02% to *Devosia submarina* (NR114333.1)	XD41-J11 (OP598872)	100.00% to *Bacillus altitudinis* (NR042337.1)
XD26-G2 (OP594395)	99.63% to *Stenotrophomonas maltophilia* (NR041577.1)	XD41-G1 (OP598861)	100.00% to *Streptomyces griseorubens* (NR041066.1)
XD26-G3 (OP594396)	99.93% to *Olivibacter soli* (NR041503.1)	XD41-G3 (OP598862)	99.56% to *Pseudomonas marincola* (NR041592.1)
XD26-G4 (OP594397)	99.50% to *Pseudorhizobium halotolerans* (NR125632.1)	XD41-G4 (OP598863)	99.71% to *Planococcus chinensis* (NR042259.1)
XD26-G5 (OP594398)	99.57% to *Pantoea dispersa* (NR116797.1)	XD41-G5 (OP598864)	99.57% to *Sphingomonas sanxanigenens* (NR121736.1)
XD26-G6 (OP594399)	98.42% to *Microbacterium thalassium* (NR042481.1)	XD41-G6 (OP598865)	99.71% to *Thalassospira xianhensis* (NR116127.1)
XD26-G7 (OP594400)	99.44% to *Paenibacillus pabuli* (NR040853.1)	XD41-G7 (OP598866)	99.85% to *Nocardioides albus* (NR036914.1)
XD26-G8 (OP594401)	98.89% to *Pseudomonas alcaligenes* (NR114472.1)	XD41-G8 (OP598867)	98.53% to *Microbacterium thalassium* (NR042481.1)
XD26-G9 (OP594402)	99.50% to *Agrobacterium larrymoorei* (NR026519.1)	XD41-G9 (OP603114)	99.19% to *Paenibacillus polymyxa* (NR114810.1)
XD29-Y1 (OP594424)	99.64% to *Stenotrophomonas maltophilia* (NR041577.1)	XD41-G10 (OP603112)	100.00% to *Arthrobacter oryzae* (NR041545.1)
XD29-Y2 (OP594425)	99.93% to *Brucella anthropi* (NR074243.1)	XD41-G11 (OP603113)	98.50% to *Microbulbifer pacificuse* (NR115928.1)
XD29-J2 (OP594416)	98.65% to *Rhizobium oryziradicis* (NR156856.1)	XD41-G13 (OP603116)	100.00% to *Brucella lupini* (NR042911.1)
XD29-J5 (OP594417)	99.58% to *Paenibacillus polymyxa* (NR114810.1)	XD41-G14 (OP603115)	99.16% to *Pseudoxanthomonas wuyuanensis* (NR126229.1)
XD29-J6 (OP594418)	99.93% to *Leclercia adecarboxylata* (NR104933.1)	XD41-G15 (OP614941)	99.71% to *Curtobacterium plantarum* (NR104943.1)

Note: XD is Xindong; Y is the leaf; J is the stem; G is the root.

**Table A2:** Species diversity of endophytic bacteria in different tissues of wheat.

Classification	Number of Endophytic Bacteria				
	Leaf	Stem	Root	Sum	Account for (%)
*Arthrobacter* sp.	1		1	2	1.47
*Achromobacter* sp.		1		1	0.73
*Agrobacterium* sp.		7	4	11	8.09
*Aurantiacibacter* sp.		1		1	0.73
*Brucella* sp.	1		1	2	1.47
*Bacillus* sp.		5	7	11	8.09
*Ciceribacter* sp.		1		1	0.73
*Curtobacterium* sp.			1	1	0.73
*Devosia* sp.		1		1	0.73
*Enterobacter* sp.		1	1	2	1.47
*Fictibacillus* sp.		1		1	0.73
*Janibacter* sp.	1			1	0.73
*Kocuria* sp.		1	1	2	1.47
*Kosakonia* sp.		1		1	0.73
*Leclercia* sp.		1		1	0.73
*Lactococcus* sp.		1	1	2	1.47
*Microbacterium* sp.	5	3	5	13	9.56
*Massilia* sp.	1			1	0.73
*Microbulbifer* sp.			2	2	1.47
*Nocardioides* sp.			1	1	0.73
*Olivibacter* sp.			1	1	0.73
*Pseudomonas* sp.	1	13	15	29	21.32
*Pseudoxanthomonas* sp.			1	1	0.73
*Pseudorhizobium* sp.			1	1	0.73
*Paenibacillus* sp.	1	2	10	13	9.56
*Planococcus chinense* sp.			1	1	0.73
*Pantoea* sp.		3	2	5	3.68
*Priestia* sp.			1	1	0.73
*Rhizobium* sp.		1		1	0.73
*Stenotrophomonas* sp.	2	3	3	8	5.89
*Staphylococcus* sp.	1	2		3	2.21
*Sphingobium* sp.		1		1	0.73
*Sphingomonas* sp.	1	1	1	3	2.21
*Salmonella* sp.		2		2	1.47
*Shinella* sp.		1		1	0.73
*Streptomyces* sp.			2	2	1.47
*Thalassospira* sp.			4	4	2.94
*Variovorax* sp.			1	1	0.73

**Table A3:** Inhibition of germination of urediniospores by endophytic bacterial strains from wheat.

Endophytic Bacteria	Inhibition Rate of CS (%)	Inhibition Rate of CSS (%)	Endophytic Bacteria	Inhibition Rate of CS (%)	Inhibition Rate of CSS (%)	Endophytic Bacteria	Inhibition Rate of CS (%)	Inhibition Rate of CSS (%)
XD5-Y1	64.93	54.51	XD22-G3	82.99	85.42	XD32-G8	74.65	60.42
XD5-Y2	70.14	77.08	XD22-G4	81.60	75.00	XD32-G9	65.63	31.25
XD5-Y3	75.35	57.29	XD26-Y2	76.74	68.40	XD32-G10	86.11	60.76
XD5-J1	92.71	87.85	XD26-J1	78.82	19.10	XD32-G11	67.71	73.61
XD5-J2	75.69	62.15	XD26-J3	25.69	67.01	XD32-G13	56.25	18.75
XD5-G2	89.24	72.22	XD26-J4	80.56	47.92	XD35-J2	74.65	77.08
XD5-G3	85.07	73.61	XD26-J5	78.13	68.40	XD35-J3	85.76	69.10
XD5-G5	79.51	70.14	XD26-G2	73.26	26.39	XD35-J4	83.68	59.38
XD5-G6	72.57	83.68	XD26-G3	67.01	48.26	XD35-J5	58.33	81.25
XD5-G7	85.76	87.50	XD26-G6	44.44	61.81	XD35-J7	73.61	51.39
XD5-G9	81.25	80.56	XD26-G7	70.14	71.53	XD35-J8	15.97	53.13
XD5-G10	67.36	70.14	XD26-G9	63.19	75.69	XD35-J9	70.14	42.71
XD5-G11	46.18	59.72	XD29-Y1	75.69	73.61	XD35-G1	73.26	74.65
XD9-Y1	54.51	84.03	XD29-Y2	82.64	72.22	XD35-G2	79.86	73.61
XD9-Y2	61.46	66.32	XD29-J2	71.18	57.99	XD35-G4	71.53	84.72
XD9-J1	75.00	42.36	XD29-J5	81.25	48.26	XD41-Y1	77.78	49.65
XD9-J2	75.35	64.93	XD29-J6	60.76	78.47	XD41-Y2	80.56	26.04
XD9-J4	79.86	70.49	XD29-J7	79.51	79.51	XD41-J1	82.64	76.04
XD9-J5	74.31	52.78	XD29-J8	69.79	70.49	XD41-J3	78.82	64.24
XD9-J6	80.56	34.38	XD29-J9	74.31	43.75	XD41-J4	67.36	64.24
XD9-J7	54.86	71.88	XD29-J10	68.75	36.11	XD41-J5	50.00	34.72
XD9-J9	85.42	75.00	XD29-J11	61.81	77.08	XD41-J6	45.83	47.92
XD9-G1	77.08	76.39	XD29-G1	84.72	86.46	XD41-J7	78.13	63.19
XD9-G2	17.71	10.42	XD29-G2	74.31	91.67	XD41-J8	87.15	80.21
XD9-G4	77.08	77.78	XD29-G3	64.93	76.04	XD41-J9	81.94	62.15
XD9-G5	77.08	38.19	XD29-G5	78.82	83.33	XD41-J10	62.50	65.97
XD14-J3	89.24	77.78	XD29-G6	82.64	61.11	XD41-J11	82.29	63.89
XD14-J4	44.44	73.96	XD29-G7	65.97	76.04	XD41-G3	76.39	34.03
XD14-J5	76.74	81.94	XD29-G8	77.43	86.81	XD41-G4	51.04	43.75
XD14-G2	85.42	45.14	XD32-Y3	54.51	31.60	XD41-G5	61.46	61.11
XD14-G3	80.90	83.68	XD32-J2	60.76	59.03	XD41-G6	62.15	64.24
XD14-G4	79.51	53.13	XD32-J3	77.78	45.83	XD41-G7	73.96	50.00
XD14-G5	64.93	59.03	XD32-J5	63.89	39.93	XD41-G8	75.69	47.22
XD14-G6	77.43	79.51	XD32-J6	86.81	80.21	XD41-G9	75.35	90.63
XD14-G8	69.79	69.10	XD32-G4	80.21	63.89	XD41-G10	76.39	40.97
XD22-J3	81.25	46.88	XD32-G5	83.68	60.76	XD41-G13	69.10	30.21
XD22-G2	61.11	57.29	XD32-G7	4.51	5.21	XD41-G15	77.08	55.56

Note: XD is Xindong; Y is the leaf; J is the stem; G is the root; CS is the culture solution; CSS is the centrifuged supernatant of the culture solution.
